# Dual Monogenic Cystic Disease Case Report: Autosomal Dominant Polycystic Kidney Disease and Autosomal Dominant Polycystic Liver Disease

**DOI:** 10.1002/ccr3.71994

**Published:** 2026-02-06

**Authors:** Anna Katya Brossart, Kathryn Curry, Sumit Punj, Tarek Darwish, Hossein Tabriziani

**Affiliations:** ^1^ Natera, Inc Austin Texas USA; ^2^ Kansas City Kidney Specialists Overland Park Kansas USA

**Keywords:** autosomal dominant polycystic kidney disease, autosomal dominant polycystic liver disease, genetic testing, polycystic kidney disease, polycystic liver disease, renal disease panel

## Abstract

Autosomal dominant polycystic kidney disease (ADPKD) and autosomal dominant polycystic liver disease (ADPLD) are inherited cystic conditions with overlapping features but distinct genetic causes and clinical courses. Here, we report a case of a 50‐year‐old woman with a clinical diagnosis of ADPKD, hypertension, preserved kidney function, significant abdominal distention consistent with hepatomegaly, and innumerable kidney and hepatic cysts. Family history was remarkable for ADPKD clinical diagnosis in the patient's mother, maternal grandmother, and the grandmother's siblings. Genetic testing with a 385‐gene NGS‐based kidney disease panel (the Renasight test) identified heterozygous truncating pathogenic variants in *PKD1*: c.3957_3994dup p.(Asp1332Glyfs*27)) (ClinVar ID VCV003376509.1) and *PRKCSH*: c.374_375del p.(Glu125fs)) (ClinVarID VCV001048653.34). To our knowledge, this is the first reported case of dual monogenic drivers of ADPKD and ADPLD in a single individual. This report highlights the importance of using unbiased genetic testing in cystic disease evaluation, even when family history suggests a single condition, to inform prognosis, reproductive risk, and accurate cascade testing in relatives.

## Key Clinical Message

A case with rare dual diagnosis of autosomal dominant polycystic kidney disease (ADPKD) and autosomal dominant polycystic liver disease (ADPLD) with a family history of ADPKD demonstrating the importance of broad renal genetic testing despite clear family history, as it can uncover rare dual monogenic drivers that can have a high degree of overlap.

## 1 Introduction

**FIGURE 1:**
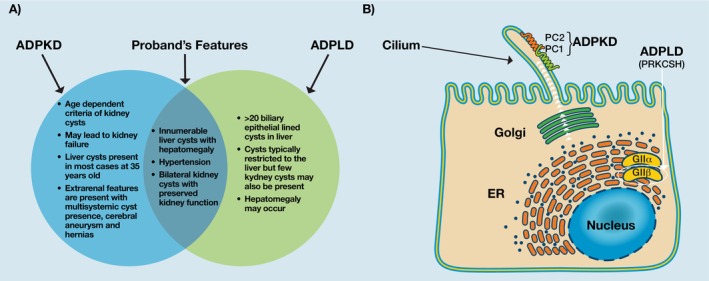
(A) Overlap of the PLD and ADPKD phenotypes. (B) Functions of the *PKD1* and *PRKCSH* gene products. *PKD1* and *PKD2* encode PC1 and PC2, respectively, which are expressed in the cilia of endothelial cells and promote signaling that involves cell growth and differentiation. *PRKCSH* encodes GII‐beta which functions in the endoplasmic reticulum (ER) to promote the maturation and secretion of proteins, including PC1 and PC2, to the cell surface. (Figure 1B is reproduced from Masyuk TV, Masyuk AI, LaRusso NF. Polycystic liver disease: Advances in understanding and treatment. Annual Review of Pathology: Mechanisms of Disease. 2006; 17: 251–269. Annual Reviews. Used with permission; permission conveyed through Copyright Clearance Center Inc).

Autosomal dominant polycystic kidney disease (ADPKD) and autosomal dominant polycystic liver disease (ADPLD) are hereditary cystic conditions with considerable phenotypic overlap but distinct genetic etiologies and natural histories (Figure 1A). ADPKD is most commonly caused by variants in the *PKD1* or *PKD2* genes, which encode the polycystin complex PC1:PC2 expressed in the cilium of endothelial cells in the kidneys and liver [1]. The *PRKCSH* gene, which is implicated in ADPLD, encodes hepatocystin, the non‐catalytic β‐subunit of glucosidase II (GII β) [2]. Glucosidase II is involved in folding processing and secretion of proteins, including PC1 and PC2, to the cell surface (Figure 1B) [3].

Typically, *PKD1* variants cause severe polycystic kidney disease (PKD), of which kidney failure is a common outcome [4]. Liver cysts may or may not be present in ADPKD, but if present, individuals can experience mild to severe PLD. The vast majority (~94%) of patients with ADPKD who develop symptomatic PLD are women [5].

*PRKCSH* variants typically cause mild‐to‐severe polycystic liver disease (PLD), with absent‐to‐mild polycystic kidney disease (PKD). PLD, of which ADPLD is the inherited form, is clinically diagnosed based on the presence of > 20 hepatic cysts, although a finding of ≥ 10 cysts should prompt consideration of PLD [2]. In the context of familial ADPLD, a threshold of ≥ 4 cysts applies [6]. In individuals with ADPLD, kidney cysts are typically few and non‐progressive. Liver cysts are asymptomatic and do not require treatment in 95%–98% of cases [7]. In the remaining ~2%–5% of patients, hepatomegaly is present with variable kidney function [8].

Here we describe a dual molecular diagnosis of ADPKD‐*PKD1* and ADPLD‐*PRKCSH* in a female with multigenerational ADPKD presenting with pronounced abdominal distention consistent with hepatomegaly. To our knowledge, no other report of an individual with dual monogenic drivers of ADPKD and ADPLD has been published. Identification of this rare dual genetic etiology has implications for prognosis, clinical management, and accurate genetic counseling for the patient and at‐risk relatives. This report underscores the benefit of an unbiased approach to genetic testing for kidney and related disorders, even in the context of a well‐established clinical diagnosis of ADPKD.

## 2 Case History/Examination

**FIGURE 2:**
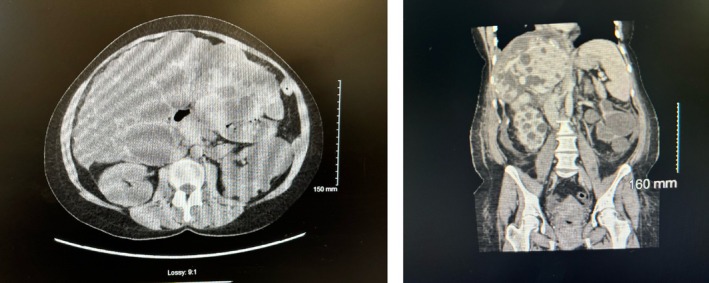
CT Scan images of the proband showing liver and kidney cysts.

A 50‐year‐old female of European descent with a clinical diagnosis of ADPKD and preserved renal function sought medical attention for abdominal discomfort. She presented to nephrology with significant abdominal distension consistent with hepatomegaly. Imaging revealed multiple kidney and hepatic cysts (Figure 2). She reported increasing discomfort due to the hepatomegaly and difficulty eating.

The patient's family history is notable for a clinical diagnosis of ADPKD in her mother, maternal grandmother, and the grandmother's siblings. Her mother experienced kidney failure secondary to ADPKD in her late 60s and received a kidney transplant; she died of complications of COVID‐19 in her 70s. Imaging performed near the end of life revealed several small liver cysts. The patient's maternal grandmother died from complications of ADPKD at 82. Another first‐degree relative of the proband has several kidney cysts with normal kidney function. There was no known family member with significant hepatic features of ADPKD or clinical diagnosis of PLD.

## 3 Differential Diagnosis

Genetic testing to identify the etiology of ADPKD in the family had not been previously performed. As the patient's presentation (i.e., significant hepatic involvement with preserved renal function) was inconsistent with the disease progression reported in other family members, broad‐panel renal genetic testing was ordered. A 385‐gene panel (the ^1^Renasight test), which includes multiple genes associated with cystic disease was used to determine the genetic etiology as described previously [9]. This gene panel was developed to test for genomic DNA variation from the patient's blood. A customized hybrid capture enrichment technology was used to sequence the coding exons, including the intron‐exon boundaries, of 385 clinically‐validated genes associated with inherited kidney disease. Paired‐end, massively parallel sequencing of 150‐bp reads was performed on the enriched DNA libraries. The sequencing data were aligned to the reference assembly, GRCh37/hg19 build. A clinically validated and in‐house developed bioinformatics pipeline based on the best practice workflow of the Genome Analysis Toolkit [10] was used for secondary analysis and variant calling. Variants were filtered and annotated using the Human Genome Variation Society (HGVS) nomenclature [11]. Variant classification was performed according to the American College of Medical Genetics and Genomics/Association of Molecular Pathology (ACMG/AMP) guidelines [12]. Confirmatory testing of the NGS‐detected variants was performed on the original DNA sample by an orthogonal method using Sanger sequencing or MLPA/qPCR as appropriate.

## 4 Conclusions and Results

Heterozygous variants in *PKD1* and *PRKCSH* that were classified as pathogenic according to the ACMG/AMP guidelines [12] and annotated according to the HGVS nomenclature [11] as NM_001009944.3 (*PKD1*): c.3957_3994dup p.(Asp1332Glyfs*27) (ClinVar ID VCV003376509.1) and NM_001289104.2 (*PRKCSH*): c.374_375del p.(Glu125fs) (ClinVarID VCV001048653.34), were identified in genes associated with ADPKD and ADPLD, respectively; both variants are predicted to be truncating.

Typically, truncating *PKD1* variants cause severe PKD and can present with mild‐to‐severe PLD. The *PKD1* variant identified here was a 38‐base‐pair duplication resulting in a frameshift in exon 15 that is predicted to lead to an out‐of‐frame transcript and the introduction of a premature termination codon (PTC). This PTC is located at least 50 nucleotides upstream of the canonical donor splice site at the penultimate exon and is consistent with the resulting transcript being targeted for nonsense mediated decay (NMD), a protective mechanism whereby expression of the abnormal protein product is greatly reduced [12, 13, 14, 15]. This variant is absent from the Genome Aggregation Database (gnomAD V4.1.0) dataset [16] and has been previously reported in the literature [17]. Due to the strong maternal family history of ADPKD, this *PKD1* variant is assumed to be maternally inherited, although this cannot be confirmed.

The patient's *PRKCSH* variant was a 2‐base‐pair deletion causing a frameshift in exon 6. The identified *PRKCSH* variant is expected to lead to an out‐of‐frame transcript and the introduction of a PTC, which will also result in NMD. This *PRKCSH* variant has been previously identified in individuals from two families with ADPLD [18, 19].

## 5 Discussion

Despite the significant phenotypic overlap between ADPKD and ADPLD, clinicians often employ genetic testing specific to either renal or hepatic cystic disease, which can fail to capture an unexpected genetic etiology. Condition‐specific testing may seem a particularly sound choice in the context of multigenerational disease, with the goal being to molecularly confirm what is already suspected. However, this report highlights the value of unbiased genetic testing for cases clinically diagnosed with ADPKD with present or suspected PLD. In this case, genetic testing was performed using a broad multi‐gene panel that covers both major and minor kidney and liver cystic disease genes rather than a single gene approach. This broad‐spectrum NGS strategy allows simultaneous detection of variants across multiple genes, enabling identification of additional variants that may contribute to disease severity. Importantly, the 2025 KDIGO ADPKD guidelines [20] strongly supports the use of a broad approach that encompasses both major and minor cystic disease genes associated with ADPKD, ADPLD, as well as other genes associated with kidney cysts. The panel applied here (the Renasight test) aligns with this guidance, covering the recognized ADPKD and ADPLD genes as well as other genes linked to cystic kidney disorders [20]. This is particularly crucial in cases with atypical, severe, or overlapping phenotypes, as it can clarify the underlying mechanism and improve risk assessment for family members. In this case, the atypical liver cyst burden relative to the progression of kidney disease led to increased suspicion of another genetic cause beyond *PKD1* or *PKD2*. If genetic testing had not been sufficiently broad to capture both causative genes, at‐risk family members could have been counseled inaccurately regarding recurrence risk.

The pathogenic *PKD1* variant identified here is absent from the Broad gnomAD V4.1.0 dataset, which consists of sequence data from ethnically diverse populations and serves as a reference for the general population [16], but was reported previously [17]. Novel variants in the *PKD1/PKD*2 genes are not uncommon, given the high degree of allelic heterogeneity in those genes. Often, a causal variant is unique to a single family and has not been reported in unrelated individuals. In this case, the patient's pathogenic *PRKCSH* variant has been previously identified in individuals from two families with ADPLD [18], and 2 alleles are present in gnomAD V4.1.0. Pathogenic variants in *PRKCSH* have been shown to result in abnormal hepatocystin, suggesting the ADPLD phenotype arises from loss‐of‐function (LOF) of the protein [21].

Importantly, the presence of ADPLD‐*PRKCSH* and ADPKD‐*PKD1* pathogenic variants likely confers greater risk of hepatic cyst progression, potentially due to a synergistic effect of the two variants. Hepatic cysts are thought to be caused by changes to the sensitivity of the primary cilium, a non‐motile structure present in cholangiocytes [7]. The polycystin 1 (PC1) protein, encoded by the *PKD1* gene, is a part of the PC1: PC2 complex that influences cell growth and differentiation of cilia, and is expressed in the cilium of endothelial cells in the kidneys and liver [1]. Dysfunction of the *PRKCSH* protein product, hepatocystin, can interfere with PC1 and PC2 expression [22]. As PC1 interacts with PC2, which in turn interacts with other cystic proteins essential for proper PC2 channel function [23], the dual variants could disturb the integrity of these protein interaction networks (Figure 1B).

In this case, it would be expected that the *PKD1* variant reduces PC1 production, while the additional *PRKCSH* variant could further diminish functional PC1/PC2 levels. This pronounced reduction in PC1 function could result in an aggressive phenotype consistent with a gene dosage effect [22] and potentially oligogenic inheritance [23], as seen in patients carrying additional variants in *PKD1*. The level of functional PC1 in those with a heterozygous pathogenic germline *PRKCSH* variant is a major determining factor of disease severity for hepatic cysts. Kidney epithelial cells appear to be more tolerant than cholangiocytes to reduced PC1 levels, which could explain the occurrence of isolated liver cysts in some individuals with ADPLD [3, 7, 22, 24], and the lack of significant kidney dysfunction with pronounced cystic liver features in this case. This case amplifies how such dual genetic diagnoses may interact to exacerbate clinical manifestations rather than acting independently. Notably, the combined occurrence of ADPKD and ADPLD pathogenic variants is exceptionally rare, with ADPKD affecting 1:1000 individuals [25] and ADPLD approximately affecting 1:100,000 [26], emphasizing the uniqueness and the clinical significance of this dual diagnosis presentation.

Dual genetic diagnoses can have important implications for genetic counseling. Determining who in prior generations had a given pathogenic variant can be informative with respect to prognosis and intrafamilial variability. Testing of prior generations was not feasible in this case, as the patient's parents are deceased, precluding familial segregation analysis. In the absence of familial segregation analysis as confirmatory testing in this case, this *PKD1* variant is only assumed to be maternally inherited (due to the strong maternal family history of ADPKD), and it remains unknown if the *PRKCSH* variant is *de novo* or inherited from an asymptomatic or mildly affected parent. Due to the variable expressivity and reduced penetrance associated with ADPLD, the absence of significant hepatic symptoms in previous generations is not sufficient evidence to conclude that the variant is *de novo*. It is possible that the variant was inherited but did not manifest prominently in earlier generations. Despite the lack of segregation data, this case highlights the importance of genetic counseling and cascade testing for at‐risk relatives, including any potential children. Importantly, when considering genetic testing for adult‐onset disorders in minors, care should be taken to balance the benefits of early detection and disease management against the potential ethical and psychosocial implications of testing.

This case also highlights the importance of an accurate and complete molecular diagnosis for cascade testing of at‐risk family members. Due to independent assortment and linkage equilibrium, the *PKD1* (located on chromosome 16) and *PRKCSH* (located on chromosome 19) alleles will co‐segregate randomly. Family members, however, are at high risk of inheriting one or both variants (e.g., for offspring there is a 25% chance of inheriting both variants and a 50% chance of inheriting one variant) and should be screened clinically for both diseases even in the absence of genetic testing [25]. For a dual diagnosis such as this, confirmatory molecular testing for both conditions would be strongly recommended for the patient's adult offspring and siblings, even if they are asymptomatic.

For patients with clinically diagnosed ADPKD, genomic confirmation can guide prognostic and management recommendations. Given the phenotypic overlap, molecular testing is often necessary to discriminate between ADPKD and ADPLD. At this time, there is no gene‐specific guidance available regarding prognosis or medical management for patients with ADPLD. Of note, in post‐menopausal women, severe PLD has been reported in individuals with variants in the cystic disease genes *PKD1, PKD2, PRKCSH*, and *SEC63*, with a suggestion of strong hormonal influence on the liver phenotype [27, 28]. High estrogen exposure resulting from pregnancy, oral contraceptive use, and estrogen‐replacement therapy is associated with more severe PLD; therefore, avoidance of estrogen‐ and progesterone‐containing oral contraceptives or intrauterine devices is recommended for women with PLD [2].

In conclusion, genetic testing with a targeted panel based on the suspected origin of the disease (i.e., hepatic or renal) would have been inadequate to capture the unexpected genetic etiology of the patient's phenotype. As this case demonstrates, unbiased genetic testing with a comprehensive panel for kidney disorders that includes all major and minor genes associated with ADPLD and ADKPD has value even in cases with established multi‐generational ADPKD. Additional functional studies of the effect of dual ADPKD/ADPLD diagnosis are needed to elucidate the mechanism of cystogenesis and the potential implications for prognosis and clinical management.

## Author Contributions

**Anna Katya Brossart:** conceptualization, supervision, writing – original draft, writing – review and editing. **Kathryn Curry:** conceptualization, writing – original draft, writing – review and editing. **Sumit Punj:** data curation, methodology, validation, writing – review and editing. **Tarek Darwish:** conceptualization, data curation, writing – review and editing. **Hossein Tabriziani:** conceptualization, investigation, writing – review and editing.

## Funding

The authors have nothing to report.

## Consent

The patient provided written consent for publication.

## Conflicts of Interest

A.K.B., S.P., and H.T. are full‐time employees of Natera Inc. with stocks and/or options to own stocks in the company. K.C.'s employment includes Natera Inc. (at the time of the study) with stocks or options to hold stocks in the company. T.D. declares no conflicts of interest.

## Data Availability

The data that support the findings of this study are available on request from the corresponding author. The data are not publicly available due to privacy or ethical restrictions.

## Endnotes

^1^
Renasight is a Natera‐owned trademark.
